# The CRISPR/Cas9 system forms a condensate in the yeast nucleus

**DOI:** 10.17912/micropub.biology.001039

**Published:** 2024-01-09

**Authors:** Sara Medina-Suárez, Félix Machín

**Affiliations:** 1 Unidad de Investigación, Hospital Universitario Nuestra Señora de Candelaria, Santa Cruz de Tenerife, Canary Islands, Spain; 2 Instituto de Tecnologías Biomédicas, Universidad de La Laguna, San Cristóbal de La Laguna, Canary Islands, Spain; 3 Facultad de Ciencias de la Salud, Universidad Fernando Pessoa Canarias, Las Palmas de Gran Canaria, Canary Islands, Spain

## Abstract

CRISPR/Cas9 gene editing technology has revolutionized genetic engineering. However, the nuclear dynamics of Cas9 in eukaryotic cells, particularly in the model organism *Saccharomyces cerevisiae* , remains poorly understood. Here, we constructed yeast strains expressing fluorescently tagged Cas9 variants, revealing their accumulation in the nucleus over time. Notably, Cas9 was non-uniformly distributed in the nucleoplasm during the initial hours, suggesting the formation of a condensate. This condensate often co-localizes with the nucleolus and associates the target site to its periphery. Our findings provide insights into Cas9 nuclear dynamics in yeast, advancing our understanding of CRISPR/Cas9-based genetic manipulation.

**Figure 1. Cas9 forms condensates in the yeast nucleus with the target sequence at the condensate periphery:**
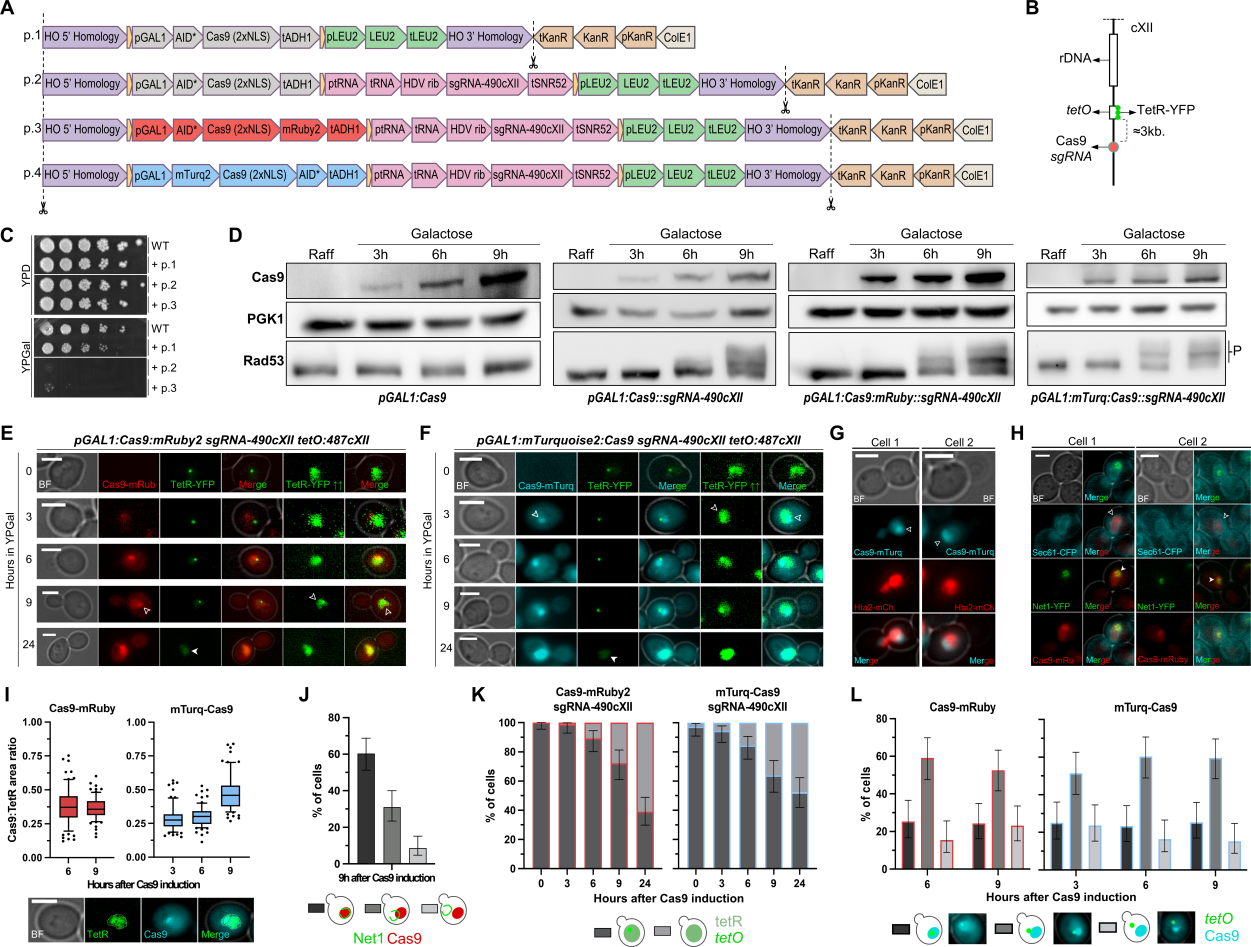
**(A)** Linear schematic of plasmids constructed using the modular cloning (MoClo) system. NotI restriction enzyme cut sites for integration at the *ho* locus are indicated by dashed lines and scissors. Plasmids are composed of: homology sequences for integration into the genome (purple); connectors (yellow); Cas9 transcriptional units (gray, red, or blue depending on the presence or absence of a Cas9-binding fluorescent protein); an sgRNA transcriptional unit (pink); a yeast selection marker transcriptional unit (green); and a bacterial marker transcriptional unit (brown) and replication origin (beige). **(B)** Schematic of the location of the sgRNA target site for Cas9 ( *490cXII* ) and its relative position to *tetO* at *487cXII* . **(C)** Spot assay on YPD and YPGal plates of a wild-type (WT; FM1249) strain and its derived isogenic strains with Cas9 under the control of the galactose promoter. Note that the strain in which plasmid 1 (p.1) was integrated does not have the sgRNA transcriptional unit. **(D)** Western blot confirming Cas9 expression after switching the carbon source from raffinose to galactose and Cas9-mediated DNA damage (Rad53 hyperphosphorylation; “-P” bands). PGK1 is used as an internal loading control. **(E)** Representative cells expressing Cas9-mRuby during the time course galactose induction. The *cXII(487Kb)* position is spotted by the *tetO* /TetR-YFP system. The nucleoplasm is labeled by the unbound pool of TetR-YFP (overexposed images are indicated by two up arrows). Hollow arrowheads point to examples where Cas9 condensates in a nucleoplasm subregion. Filled arrowhead highlights examples where the *tetO* signal is lost (24h in galactose). **(F)** As in (E), but in cells expressing mTurquoise-Cas9. **(G)** Two representative cells of mTurquoise-Cas9 condensates after 9 hours in YPGal relative to the nuclear DNA mass labeled with the histone Hta2-mCherry. The arrowhead points to the Ca9 condensate **(H)** Two representative cells of Cas9-mRuby condensates 9 hours after induction relative to the nuclear envelope marker Sec61-eCFP and the nucleolar marker Net1-eYFP. In both cells, the filled arrowhead highlights that Cas9 occupies only a fraction of the area defined by Sec61. The hollow arrowheads indicate that Cas9 and Net1 colocalize perfectly in cell 1, yet only partially in cell 2. **(I)** Quantification of the fraction of nucleoplasm covered by Cas9 (represented as Cas9:TetR ratio). A descriptive example with the area occupied by each signal marked and superimposed is shown below the graphs. **(J)** Quantification of the colocalization between the Cas9 condensate and the nucleolar marker Net1. Schematics of the different categories are depicted below. **(K)** Quantification of *tetO* loss after Cas9 induction in YPGal. The strain expressing Cas9-mRuby plus *sgRNA-490cXII* is on the left and mTurq-Cas9/ *sgRNA-490cXII* is on the right . The control without sgRNA had >98% cells with *tetOs* after 24h in YPGal. **(L)** Quantification of *tetO:487* position relative to Cas9 condensate. Dark gray bars indicate that the *tetO* is surrounded by Cas9, medium gray when the tetO touches the periphery of the Cas9 condensate, and light gray when the *tetO* is outside the Cas9 condensate (examples shown below). In photomicrographs, the scale bar corresponds to 3 microns. BF, bright field.

## Description

The advent of the Clustered Regularly Interspaced Short Palindromic Repeats (CRISPR) and CRISPR-associated protein 9 (Cas9) system has deeply transformed genetic engineering and genome editing, enabling precise and targeted modifications of DNA sequences in a wide range of organisms (Doudna and Charpentier 2014) . Cas9 is an endonuclease responsible for generating DNA double-strand breaks at specific target sites defined by a short guide RNA (sgRNA). However, while Cas9 has been extensively employed for genome editing, much remains to be elucidated regarding its intracellular behavior and dynamic localization within eukaryotic cells. Microscopy techniques have been invaluable for visualizing the spatiotemporal distribution of proteins within living cells. Studies of Cas9 localization in mammalian cells have been mostly focused on its use to visualize specific loci. In this context, an endonuclease-dead mutant (dCas9) is used, and the distribution includes a pool of dCas9 bound to the target sequence(s) together with another pool that is uniformly distributed throughout the nucleoplasm but enriched at nucleoli (Chen et al., 2013) . However, this pattern is not universal as, for example, only the target-bound pool is present in *Caenorhabditis elegans* embryos (Memar et al., 2022) . Of note, the localization of Cas9 in *Saccharomyces cerevisiae* cells, a classical model organism for eukaryotic genetics, remains comparatively less explored and, to the best of our knowledge, has only been studied in relation to the optimization of nucleocytoplasmic shuttling strategies (Roggenkamp et al., 2018) . Now, we have addressed this crucial knowledge gap by a time course exploration of Cas9 localization within the yeast nucleus.

To this end, we constructed four yeast strains expressing yeast-optimized Cas9 variants assembled using the Golden Gate modular cloning (MoClo) method (Lee et al., 2015) . One strain expresses only the basic Cas9 protein under the inducible *GAL1* promoter ( Figure 1A, p . 1). A second strain also co-expresses an sgRNA against a genomic region 3 Kb away from a *tetO* array inserted at the 487 Kb coordinate in chromosome XII ( *sgRNA-490cXII* ) ( Figure 1A, p .2; Figure 1B ). This *tetO* array is a tool to detect this particular genomic region as a spot through its specific binding to the TetR reporter, which in turn is fused to the fluorescent protein YFP (yellow/green fluorescence) (Machín et al., 2005). Importantly, not all TetR-YFP molecules bind the *tetO* array; an unbound pool freely and uniformly circulates in the nucleus. The third strain is a derivative of the latter in which Cas9 is fused at the C-terminus to the fluorescent protein mRuby2 (red fluorescence) ( Figure 1A, p . 3), while the fourth strain has the fluorescent protein mTurquoise2 (cyan fluorescence) fused to the N-terminus of Cas9 ( Figure 1A, p . 4). Functional analysis by spot assays showed that co-expression of Cas9 constructs and *sgRNA-490cXII* completely inhibited cell growth ( Figure 1C, p .2 or p.3 in YPgal), whereas neither Cas9 alone (p.1 in YPgal) nor *sgRNA-490cXII* alone (p.2 or p.3 in YPD) were toxic. Correspondingly, evidence of Cas9-mediated DNA damage, as measured by hyperphosphorylation of the DNA damage checkpoint reporter Rad53, was only observed when the Cas9 variants were co-expressed with *sgRNA-490cXII* ( Figure 1D ). The timing of Rad53 hyperphosphorylation in a time course of Cas9 expression showed a delay of approximately 3 h relative to the appearance of Cas9 (6 h and 3 h from induction, respectively) ( Figure 1D ).

Microscopic detection of the fluorescently tagged Cas9 variants showed that Cas9-mRuby2 was not visible 3 h after Cas9 induction, although Cas9 was clearly detected by Western blot at the same time point ( Figure 1D and 1E). By contrast, there was good agreement between microscopy and Western blots for mTurquoise2-Cas9 detection ( Figure 1D and 1F). The delay in fluorescence detection of Cas9-mRuby2 is likely related to the slow maturation of red fluorescent proteins (Balleza et al., 2018) . Both Cas9-mRuby2 and mTurquoise2-Cas9 accumulated in the yeast nucleus over time, and an intense signal covered the entire nucleoplasm by 24 h ( Figure 1E and 1F). Interestingly, however, the distribution of Cas9 was not uniform during the first 9 h, with Cas9 confined to less than 25%-50% of the nucleoplasmic area, as reported by the freely circulating pool of NLS-TetR-YFP, the nuclear DNA histone Hta2, and the nuclear envelope marker Sec61 ( Figure 1E -I). The fact that Cas9 is not uniformly distributed across the nucleoplasm strongly suggests that the Cas9 sgRNA partner forms a condensate, as has been proposed for the yeast nucleolus (Lawrimore et al., 2021) . Based on this similarity and the fact that Cas9 is enriched in human nucleoli (Chen et al., 2013) , we also examined whether the Cas9 condensate associates with the yeast nucleolus. We found partial co-localization with the nucleolar marker Net1 ( Figure 1H,J ). In ~60% of cells the Cas9 condensate perfectly colocalizes with Net1, whereas in ~30% of cells Cas9 and Net1 partially co-localized. Only in ~8% of cells did Net1 and Cas9 clearly occupy distinct nuclear subdomains. Thus, there appears to be a tendency for Cas9 to condensate in the yeast nucleolus.

Finally, we asked whether the Cas9 condensate nucleates around the expected cut site in the genome. If this were the case, we would expect to see Cas9 surrounding the *tetO* /TetR spot. Note that the percentage of cells with visible spots decreased over the time course of Cas9/ *sgRNA-490cXII* co-expression ( Figure 1K ), likely as a result of resection of the Cas9-generated double-strand break (Mosbach et al., 2020) . Of those cells where the spot was visible, approximately 25% had the spot surrounded by Cas9 ( Figure 1L ). This percentage is similar to the subnuclear area covered by the Cas9 condensate and is therefore consistent with the expected percentage by chance. However, the *tetO* spot did not follow a random location in the remaining cells. In fact, in up to 60% of cells overall (~80% of cells with the *tetO* not surrounded by Cas9), the *tetO* spot is located at the periphery of the Cas9 condensate ( Figure 1L ), with no differences in distribution between Cas9-mRuby2 and mTurquoise2-Cas9. This result suggests that the genomic region to be cut may stably contact the periphery of the Cas9 condensate. However, since the cutting site was designed near the ribosomal DNA array ( Figure 1B ), further work will be required to discern the actual cause of the Cas9-nucleolus-target site association.

In conclusion, Cas9 is not homogeneously distributed in the yeast nucleoplasm, but forms a condensate that occupies one third of the nuclear area. Interestingly, this condensate does not assemble onto the target site in the genome, but around the nucleolus, although this site is attached to the condensate periphery. Our investigation fills a knowledge gap that not only advances our understanding of CRISPR/Cas9-based genetic manipulation in yeast, but may also contribute to a broader understanding of Cas9 behavior across eukaryotic species.

## Methods

**Yeast strains and plasmids**

Most yeast strains used stem from FM1249, which is derived from the YPH499 background. FM1249 carries several genetic modifications, including: (i) a fluorescent tag for the distal part of the rDNA array on the right arm of chromosome XII ( *tetO:487* /NLS-TetR-YFP system; NLS, nuclear location signal), (ii) the *cdc14-1* thermosensitive allele, which arrests cells in early anaphase at the restrictive temperature of 37 ºC (Machín et al., 2016), and (iii) an integrated construct to constitutively express the auxin-inducible degron system (OsTIR1) (Morawska and Ulrich 2013) . FM3203 and FM3205 are wild-type strains from the W303 background that carry fluorescent tags for nuclear reporters apart from expressing Cas9.

Different multigene plasmids carrying transcriptional units (TU) for expressing a yeast-optimized Cas9 under the control of the *GAL1* promoter ( *GAL1p* ) were generated by the MoClo Yeast Toolkit (Lee et al., 2015) ( Figure 1A ). The *ADH1* terminator ( *ADH1t* ) was used in all TUs. The toolkit already provides a cloning module for Cas9 as the sole open reading frame between the promoter and the terminator (type 3 module, pYTK036). In order to add tags at both the N- and C-terminus, we made a Cas9 type 3b module (see plasmid table in Reagents) by PCR using a set of primers with the corresponding tails (see primer table in Reagents). In this manner, Cas9 was fused to fluorescent proteins in the corresponding TUs ( Figure 1A ). Likewise, the minimal auxin-inducible degron ( AID* ) sequence was tagged to the second terminus of Cas9 to make Cas9 degradable. The AID* sequence, obtained from the plasmid pKan-AID*-9myc (addgene code #99522; #2188 in the Urlich collection) (Morawska and Ulrich 2013) , was converted into a 4a module by PCR, whereas an AID* 3a module was ordered as a synthetic gene (gBlocks HiFi Gene fragments, IDT). In these multigene plasmids, a second TU was assembled to include an sgRNA designed to cut at coordinate 490 Kb on the chromosome XII right arm, just 3 Kb away from the *tetO:487* fluorescent tag. Finally, the multigene cassette was designed to be flanked by sequences homologous to the *ho* locus on yeast chromosome IV. In all cases, the different transcriptional units were assembled using pYTK095 as a backbone (bacterial AmpR-ColE1 type 678 module). If the final plasmid has only one TU, it is flanked by LS and RE connectors; if it has two, the first TU is flanked by LS and R1 connectors, while the second is flanked by L1 and RE.

Integration of Cas9 variants and sgRNA was achieved by releasing the cassette by cutting with NotI-HF (NEB, R3189L) ( Figure 1A ), followed by standard transformation procedures (Knop et al., 1999) .

**Experimental conditions**

Strains were grown overnight in air orbital incubators at 25 ºC in YP media (10 g·L ^−1^ yeast extract, 20 g·L ^−1^ peptone) with raffinose 2% (w/v) as carbon source, then galactose 2% (w/v) was added to log-phase asynchronous cultures whose OD _600_ was previously adjusted to ~ 0.4. Samples were taken at different time points during 24 h for Western blotting and/or wide field fluorescence microscopy. Neither the early anaphase arrest property of the *cdc14-1* allele nor AID*-mediated conditional degradation by auxin in FM1249-based strain was tested in this work. At 25º C, Cdc14-1 is functional in this background. The W303-based strains carry a wild type *CDC14* gene.

Western blotting and microscopy were carried out as reported before (Matos-Perdomo et al., 2022) . A fully motorized Leica DMI6000B microscope with an ultra-sensitive DFC350 digital camera and a 63X/1.30 immersion objective was used for wide field fluorescence microscopy. A stack of 20 z-focal plane images (~0.2 µm depth) was collected for each field. Images were taken from freshly harvested cells and a minimum of 70 cells were quantified per experimental data point. The AF6000 (Leica) and Fiji-ImageJ (NIH) software were used for image processing and quantification. Two-dimensional maximum projections of the focal area in the z-slices were used for analysis.

For viability spot assays, cultures were adjusted to OD _600_ = 0.5 and then serially diluted 5-fold in 96-well plates. A 48-pin replica plater was used to spot ~3 µL onto the corresponding plates, which were incubated at 25 °C for 3–4 days before taking photographs.

**Data display**

Graphpad Prism 10 was used to present data as either bar graphs or box plots. For bar graphs, error bars represent 95% confidence intervals calculated using the Wilson/Brown method. In box plots, the median line represents the median, the box limits represent the 25th and 75th percentiles, the whiskers extend to the 10th and 90th percentiles, and the dots represent outliers.

## Reagents

**Table:** 

**Strain**	**Genotype**	**Origin**	**Use**
FM1249	*MAT* a *bar1Δ leu2-3,112 his3-Δ200 trp1-Δ63 lys2-801 ade2-1::TetR:YFP::ADE2 ura3-52::ADH1:OsTIR1:9Myc::URA3 ChrXII(487Kb)::tetO(x224)::HIS3::ChrXII(487Kb) cdc14-1:9myc::TRP1*	F.Machín lab	1C
FM2664	FM1249; ho::GAL1p:AID*:CAS9::LEU2::ho	This work	1C, 1D, 1K
FM2643	FM1249; ho::GAL1p:AID*:CAS9::sgRNA-490cXII::LEU2::ho	This work	1C, 1D
FM2644	FM1249; ho::GAL1p:AID*:CAS9:mRUBY2::sgRNA-490cXII::LEU2::ho	This work	1C, 1D, 1E, 1I, 1K, 1L
FM2710	FM1249; ho::GAL1p:mTURQUOISE2:CAS9:AID*::sgRNA-490cXII::LEU2::ho	This work	1D, 1F, 1I, 1K, 1L
FM3203	MATa ade2-1 his3-11,15 trp1-1 can1-100 leu2-3,112 HTA2:mCHERRY::URA3 ho::GAL1p:mTURQUOISE2:CAS9:AID*::sgRNA-490cXII::LEU2::ho	This work	1G
FM3205	Mata ade2-1 his3-11,15 trp1-1 can1-100 leu2-3,112 ura3-1::ADH1:OsTIR1:9Myc::URA3 SEC61:eCFP::kanMX4 NET1:eYFP::Hph ho::GAL1p:AID*:CAS9:mRUBY2::sgRNA-490cXII::LEU2::ho	This work	1H, 1J

**Table:** 

**Plasmid**	**Origin**	**Description**
pFM431 (p.1)	pFM427 as the single TU ( GAL1p:AID*:CAS9:tADH1 ) + pFM335 as the backbone ( *LEU2* TU; *ho* 3’ and 5’ homologies; *kanR-ColE1* )	MoClo plasmid to insert *GAL1p:CAS9* at the *ho* locus
pFM432 (p.2)	pFM428 as the first TU ( GAL1p:AID*:CAS9:tADH1 ) + pFM357 as the second TU (sgRNA to cXII-490 position) + pFM335 as the backbone ( *LEU2* TU; *ho* 3’ and 5’ homologies; *kanR-ColE1* )	MoClo multigene plasmid to insert *GAL1p:CAS9* and *sgRNA-490cXII* at the *ho* locus
pFM433 (p.3)	pFM429 as the first TU ( GAL1p:AID*:CAS9:mRUBY2:tADH1 ) + pFM357 as the second TU (sgRNA to cXII-490 position) + pFM335 as the backbone ( *LEU2* TU; *ho* 3’ and 5’ homologies; *kanR-ColE1* )	MoClo multigene plasmid to insert *GAL1p:CAS9:mRUBY* and *sgRNA-490cXII* at the *ho* locus
pFM434 (p.4)	pFM430 as the first TU ( GAL1p:mTURQUOISE2:CAS9:AID*:tADH1 ) + pFM357 as the second TU (sgRNA to cXII-490 position) + pFM335 as the backbone ( *LEU2* TU; *ho* 3’ and 5’ homologies; *kanR-ColE1* )	MoClo multigene plasmid to insert *GAL1p:mTURQ2:CAS9* and *sgRNA-490cXII* at the *ho* locus
pFM422	CAS9-3b-stop PCR product inserted in the pYTK001 part plasmid	Part Plasmid with *CAS9* 3b ending in a stop codon
pFM423	CAS9-3b-nostop PCR product inserted in the pYTK001 part plasmid	Part Plasmid with *CAS9* 3b without the stop codon
pFM424	sgRNA-CXII490-F/R dsDNA inserted in the pYTK050 plasmid	RNA pol III transcriptional unit for *sgRNA-490cXII*
pFM425	Synthetic AID* 3a gene inserted in the pYTK001 part plasmid	Part Plasmid carrying AID* 3a
pFM426	AID-4a PCR product inserted in the pYTK001 part plasmid	Part Plasmid carrying AID* 4a

**Table:** 

**Primer**	**Sequence (5’→3’)**
Cas9-3b-F	GCATCGTCTCATCGGTCTCATTCTGACAAGAAGTATTCTATCGGACTG
Cas9-3b-R-stop	ATGCCGTCTCAGGTCTCAGGATCAGGATCCTTATACCTTTCTCTTC
Cas9-3b-R-nostop	TGCCGTCTCAGGTCTCAGGATCCTACCTTTCTCTTCTTTTTTGGATCTACC
sgRNA-cXII490-F	GACTTTCCGCCCCAGCCAAACTCTCC
sgRNA-cXII490-R	AAACGGAGAGTTTGGCTGGGGCGGAA
AID-4a-F	GCATCGTCTCATCGGTCTCAATCCCCTAAAGATCCAGCCAAACCTC
AID-4a-R	ATGCCGTCTCAGGTCTCAGCCATTACTTCACGAACGCCGCCGC

**Table:** 

**Antibody**	**Type**	**Animal and Clonality**	**Supplier**	**Reference**
anti-Pgk1	Primary	Mouse monoclonal	Thermo Fisher Sci.	22C5D8
anti-Rad53	Primary	Mouse monoclonal	Abcam	ab166859
anti-CRISPR-Cas9	Primary	Rabbit monoclonal	Abcam	ab189380
anti-Mouse (HRP)	Secondary	Goat polyclonal	Promega	W4021
anti-Rabbit (HRP)	Secondary	Goat polyclonal	Abcam	ab97051
